# Nano-electromechanical spatial light modulator enabled by asymmetric resonant dielectric metasurfaces

**DOI:** 10.1038/s41467-022-33449-9

**Published:** 2022-10-03

**Authors:** Hyounghan Kwon, Tianzhe Zheng, Andrei Faraon

**Affiliations:** 1 T. J. Watson Laboratory of Applied Physics and Kavli Nanoscience Institute, California Institute of Technology, 1200 E. California Blvd., Pasadena, CA 91125 USA; 2 Department of Electrical Engineering, California Institute of Technology, 1200 E. California Blvd., Pasadena, CA 91125 USA

**Keywords:** Nanophotonics and plasmonics, NEMS, Metamaterials, Nanoscale materials

## Abstract

Spatial light modulators (SLMs) play essential roles in various free-space optical technologies, offering spatio-temporal control of amplitude, phase, or polarization of light. Beyond conventional SLMs based on liquid crystals or microelectromechanical systems, active metasurfaces are considered as promising SLM platforms because they could simultaneously provide high-speed and small pixel size. However, the active metasurfaces reported so far have achieved either limited phase modulation or low efficiency. Here, we propose nano-electromechanically tunable asymmetric dielectric metasurfaces as a platform for reflective SLMs. Exploiting the strong asymmetric radiation of perturbed high-order Mie resonances, the metasurfaces experimentally achieve a phase-shift close to 290^∘^, over 50% reflectivity, and a wavelength-scale pixel size. Electrical control of diffraction patterns is also achieved by displacing the Mie resonators using nano-electro-mechanical forces. This work paves the ways for future exploration of the asymmetric metasurfaces and for their application to the next-generation SLMs.

## Abstract (web summary)

This work experimentally demonstrates nano-electromechanically tunable asymmetric dielectric metasurfaces. The metasurfaces enable large phase tuning, high reflection, a wavelength-scale pixel size, and electrical control of diffraction patterns.

## Introduction

Spatial light modulators (SLMs) enable spatiotemporal control of phase, amplitude, or polarization of input free-space light. In particular, phase-dominant SLMs realize efficient wavefront engineering and play essential roles in various applications such as LIDAR^1^, holographic display^2^, optical computing^3^, and bio-imaging^4,5^. Most conventional SLMs rely on liquid crystals or micromechanical systems, which result in low speed and limited field of view^6^. Recently, metasurfaces have emerged as diffractive optical elements where the pixel size is on the scale of a wavelength^7–9^. Achieving similar spatial resolution but with an active metasurface would enable high-speed and high-resolution SLMs in a compact footprint^10^. Many seminal works have been proposed in the context of the active metasurfaces, using various active materials or mechanisms such as transparent conducting oxides^1,11,12^, liquid crystals^13^, electro-optic materials^14–17^, phase-change materials^18^, 2D materials^19–22^, electromechanical systems^23–28^, and semiconductors^29^. The active plasmonic metasurfaces based on transparent conducting oxides^1,11,12^ have successfully achieved complex modulation. However, as they operate near the epsilon-near-zero regime where the light is critically coupled to lossy plasmonic resonant modes, most of the light is absorbed when a large phase shift occurs so the efficiency is significantly limited^1^. Regarding the photonic structures, most of the reflective active metasurfaces exploit mirrors to achieve strong phase response at optical resonances^1,11,12,18,20,29^. The presence of the mirror ensures that the radiation of the resonance is matched with the input light, enhancing the phase response in reflection^30^. However, including a mirror in the structure complicates fabrication, and a way to achieve strong phase shift and high reflection without the mirror has remained illusive.

Here, we propose nano-electromechanically tunable asymmetric metasurfaces to realize phase-dominant SLMs. In particular, by exploiting asymmetric high-Q Mie modes and nano-electromechanical system (NEMS), the active metasurfaces operate as efficient reflective SLMs without mirrors. First, we provide an analytical model that not only describes the physical picture of the proposed system, but also offers design intuitions. Then we numerically and experimentally verify that the proposed metasurfaces achieve strong phase modulation, high reflection, and a wavelength-scale pixel size. Finally, we experimentally demonstrate electrically controllable diffraction.

## Results

### Theoretical and numerical study of asymmetric resonant metasurfaces

**Fig. 1:**
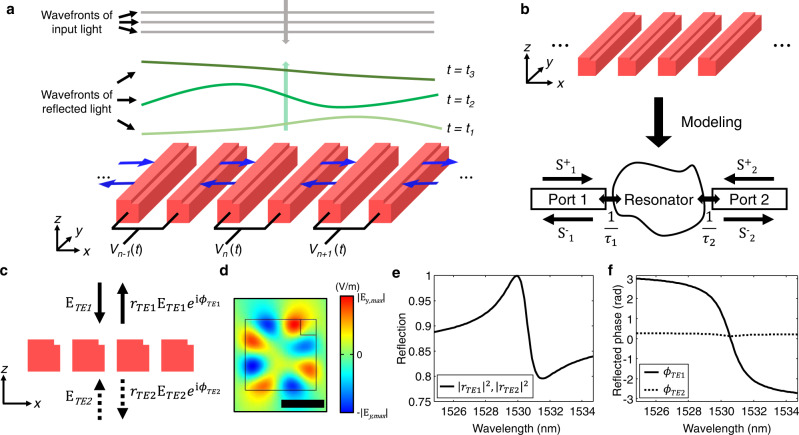
Conceptual schematic of nano-electromechanically tunable asymmetric metasurfaces and numerical investigations of their resonant reflection behavior. **a** Conceptual illustration of the nano-electromechanically tunable metasurfaces composed of asymmetric suspended nano-beams. Each pair of nano-beams is connected to individually addressable electrodes. The electrical biases induce electrostatic forces between the neighboring bars, leading to lateral movements along the *x* axis (see blue arrows). The asymmetric metasurface reflects normally an incident plane wave and dynamically manipulates the wavefront of the reflected light as a function of the applied biases. *t*_1_, *t*_2_, and *t*_3_ represent different electrical biasing conditions. **b** Schematic of a periodic metasurface (top) and illustration of the corresponding analytical model based on an optical resonator coupled to two ports (bottom). The model describes the asymmetric metasurface under normal incident light. ${S}_{1}^{+}$ (${S}_{2}^{+}$) and ${S}_{1}^{-}$ (${S}_{2}^{-}$) are incoming and outgoing waves through the port 1 (port 2), respectively. The resonance decays into the port 1 and 2 with decay rates, $\frac{1}{\tau _{1}}$ and $\frac{1}{\tau_{2}}$, respectively. **c** Schematic illustration of the asymmetric metasurfaces showing the perturbed gratings and the notches placed at the right top corners. The TE-polarized light is normally incident from either the top or the bottom side of the metasurface. ∣*r*_*TE*,1_∣ (∣*r*_*TE*,2_∣) and *ϕ*_*TE*,1_ (*ϕ*_*TE*,2_) are reflected amplitude and phase for the top (bottom) illumination, respectively. **d** Simulated electric field profile (*y*-component, 1531 nm wavelength) of the asymmetric metasurface's eigenmode at Γ point. Scale bar denotes 500 nm. **e**, **f** Calculated reflection and reflected phase spectra of the asymmetric metasurfaces for top and bottom illuminations. **e** The reflection spectra are identical for both illumination conditions, thus plotted by one solid black curve. **f** Solid and dashed curves represent the reflected phase spectra for top and bottom illuminations, respectively.

Figure 1a shows a conceptual illustration of the proposed metasurface. The metasurface consists of suspended silicon (Si) nano-bars with rectangular cross-sections perturbed with notches at one of the corners. Furthermore, each pair of nano-bars is connected to an electrode enabling its lateral movement^27^. As a result, the metasurface actively manipulates the wavefronts of reflected light as a function of the applied biases.

First, we model the suspended metasurface with temporal coupled mode theory (TCMT) to get a rigorous physical picture of the system as well as design intuition^30,31^. As shown in Fig. 1b, the metasurface under normal incidence can be generally modeled by a resonator coupled to two ports^32,33^. When driving the metasurface with a continuous laser, whose frequency is *w*, the complex reflection coefficient for each port, *r*_1_ and *r*_2_, can be derived by (see Supplementary Note 1 for detailed derivation):

1 $${r}_{1}=\frac{i\left[r(w-{w}_{0})\pm \sqrt{\frac{2}{{\tau }_{1}^{2}}+\frac{2}{{\tau }_{2}^{2}}-\frac{{r}^{2}}{{\tau }_{tot}^{2}}-\frac{1}{{r}^{2}{\sigma }^{2}}}\right]-\frac{1}{r\sigma }}{i(w-{w}_{0})+\frac{1}{{\tau }_{tot}}},$$

2 $${r}_{2}=\frac{i\left[r(w-{w}_{0})\pm \sqrt{\frac{2}{{\tau }_{1}^{2}}+\frac{2}{{\tau }_{2}^{2}}-\frac{{r}^{2}}{{\tau }_{tot}^{2}}-\frac{1}{{r}^{2}{\sigma }^{2}}}\right]+\frac{1}{r\sigma }}{i(w-{w}_{0})+\frac{1}{{\tau }_{tot}}},$$

where *r* is the real reflection coefficient of the direct scattering process; *w*_0_ is the resonant frequency; $\frac{1}{{\tau }_{1}}$ and $\frac{1}{{\tau }_{2}}$ are the resonator’s radiative decay rates into port 1 and port 2, respectively; $\frac{1}{{\tau }_{tot}}=\frac{1}{{\tau }_{1}}+\frac{1}{{\tau }_{2}}$ and $\frac{1}{\sigma }=\frac{1}{{\tau }_{1}}-\frac{1}{{\tau }_{2}}$ represent total radiative decay rate and the difference between the two radiative decay rates, respectively. In general, the coupling condition between the port and the resonator determines the phase response of the reflected light^30^. When a decay rate into a certain port is larger than the sum of other decay rates including radiative and non-radiative decay rates, the resonance is over-coupled to the port that results in almost 2*π* phase shift across the resonance frequency. In contrast, when the resonance is under-coupled to the port, the phase shift becomes negligible. To achieve high phase modulation, we aim to design an over-coupled resonator and tune its strong phase response near the resonance frequency^34^. In Eqs. (1) and (2), the coupling conditions are determined by $\frac{1}{\sigma }$. If $\frac{1}{\sigma } \, > \, 0$, the port 1 is over-coupled and the port 2 is under-coupled, and vice versa. Besides, strong asymmetric radiation makes the magnitude of $\frac{1}{r\sigma }$ comparable to the magnitude of $\frac{1}{{\tau }_{tot}}$ in Eqs. (1) and (2), leading to the decrease of the reflection loss at the resonance and the phase-dominant response^34^. Finally, the ratio between the two decay rates is bounded by the direct scattering process ^32^:

3 $$\frac{1-r}{1+r}\le \frac{{\tau }_{2}}{{\tau }_{1}}\le \frac{1+r}{1-r}.$$

Equation (3) indicates that high *r* is necessary for the desired strong asymmetric radiation. In contrast to the asymmetric cases, the symmetric resonators always cause critical coupling, resulting in negligible reflection and ~*π* phase shift near the resonance (See Supplementary Note 1 for details). The TCMT describing the asymmetric resonators has been explored previously^32,33^, and the asymmetric resonator has been recently demonstrated in experiment^35^. However, the phase responses of the asymmetric resonances have been investigated very recently by passive slanted gratings and guided mode resonances^36^. Up to our best knowledge, the asymmetric metasurfaces’ potentials in active devices have not been explored yet.

To physically implement such an asymmetric resonator, we break the symmetry of conventional gratings in Fig. 1c, making a notch at one of the top corners of each nanobar. The nano-bars are 841 nm wide, 838 nm thick and are periodically arranged with lattice constant of 1093 nm. Based on Eq. (3), the thickness, width, and lattice constant of the unperturbed structures are selected to make *r* higher than 0.9 in the telecom wavelength range. Each notch has a square cross-section with 184 nm side length. The electric field profile of the eigenmode at Γ point is shown in Fig. 1d, originating from the high-order Mie mode of an isolated Si nanobar (see Supplementary Figure 1 for details). It is worth explicitly noting why we select the high-order Mie mode instead of the guided mode. Supported by the individual nanostructure instead of the periodic lattice, the Mie mode efficiently supports wavefront shaping with the small pixel size. Specifically, in the scheme of the nano-electromechanical modulation shown in Fig. 1a, each pair of the nanostructures individually hosts the resonance and works as an independent reflective resonant antenna^7^. In addition, the notches simultaneously break the mirror symmetry in the *z*-direction as well as the even symmetry of the resonant mode under *C*_2_ rotation (180^∘^ rotation around the *z* axis). The former aims to improve phase responses through asymmetric radiations, while the latter enables coupling between the Mie mode and normally incident light. The spectra of the reflection and reflected phase are calculated for top and bottom illuminations and plotted in Fig. 1e, f. The calculated reflection spectra are identical for both illumination conditions in Fig. 1e, whereas the phase responses in Fig. 1f show strong and negligible phase responses for the top and bottom illuminations, respectively. The illumination-dependent phase responses in Fig. 1f result from the distinct coupling conditions determined by the two radiative decay rates. In particular, for the top illumination, the metasurfaces simultaneously achieve ~2*π* phase shift and high reflection over 78% over the spectrum. We also fit the simulated spectra in Fig. 1e, f by using Eqs. (1) and (2) (see Supplementary Figure 2 for details). The fitted results in Supplementary Figure 2 show good agreement with the numerical eigenmode analysis in Supplementary Figure 3. In addition to the asymmetric cases, the metasurfaces possessing mirror symmetry in the *z*-directions are investigated, showing limited phase response and negligible reflection at the resonance (see Supplementary Notes 1 and 2 and Supplementary Figure 4 for details).

### Numerical investigations on nanomechanical phase modulation

**Fig. 2:**
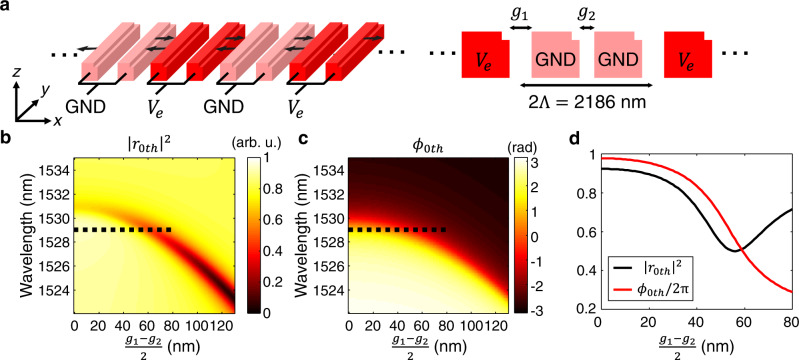
Simulations on nano-electromechanical phase modulation. **a** Schematic illustrations of an array of pairs of the asymmetric nanostructures. For every two pairs, one pair is connected to ground (GND, pink) and the other pair is connected to an external bias (*V*_*e*_, red). Left: when the external bias is applied, induced electrostatic forces result in lateral movements (see black arrows). Right: side view of the asymmetric metasurfaces is shown with design parameter definitions. **b** Calculated spectra of reflected power coefficient and phase of the 0th order diffraction, ∣*r*_0th_∣^2^ and *ϕ*_0th_. The spectra are plotted as a function of the nanomechanical tuning $\frac{{g}_{2}-{g}_{1}}{2}$. **d** Calculated nanomechanical tuning of ∣*r*_0th_∣^2^ and *ϕ*_0th_/2*π* at the wavelength of 1529 nm. ∣*r*_0th_∣^2^ and *ϕ*_0th_/2*π* are plotted by black and red curves as a function of $\frac{{g}_{2}-{g}_{1}}{2}$, respectively. The corresponding data is noted by black dashed lines in **b**, **c**.

We numerically investigate the phase modulation, utilizing the nano-electromechanical displacement in lateral directions^27,28^. Every two pairs of nano-bars, is either grounded or connected to an external bias in Fig. 2a, with *g*_1_ and *g*_2_ the gaps between nano-bars with different and same biases, respectively. The applied bias enables continuous control of the nanomechanical movement, expressed by $\frac{{g}_{1}-{g}_{2}}{2}$. To investigate the nano-electromechanical tuning, a pair of nano-bars is simulated by changing $\frac{{g}_{1}-{g}_{2}}{2}$. As the period of a pair of the nano-bars, 2Λ, is 657 nm larger than the design wavelength of 1529 nm in Fig. 2a, the induced nanomechanical movement causes unwanted diffraction orders at ±44^∘^. Nevertheless, the unwanted diffractions at ±44^∘^ become more suppressed when the structures are re-arranged for the desired wavefront engineering. The reflection power coefficient and phase of the 0th order, ∣*r*_0*th*_∣^2^ and *ϕ*_0*th*_, are calculated and plotted in Fig. 2b, c, respectively. In Fig. 2b, the blue-shift of the resonances and the decrease of the minimum reflection are observed when $\frac{{g}_{1}-{g}_{2}}{2}$ increases. The decrease of the minimum reflection dominantly results from the ±1st order diffractions (see Supplementary Figure 5 for details). In Fig. 2c, the mechanical tuning results in a continuous blue-shift of the resonance while the strong phase response remains, indicating that the phase can be modulated near the resonant wavelength. Specifically, at the wavelength of 1529 nm, ∣*r*_0*th*_∣^2^ and *ϕ*_0*th*_ are plotted in Fig. 2d as a function of the nanomechanical tuning, revealing that the nanoscale movement within 80 nm can lead to phase modulation up to 246^∘^ with minimal ∣*r*_0*th*_∣^2^ >0.47. In Fig. 2d, we set the maximum mechanical movement at 80 nm to avoid irreversible stiction of the nanostructures, which is known as the pull-in effect. Utilizing a pair of the asymmetric nanostructure as a building block, we also numerically show the metasurfaces’ capability of beam steering (see Supplementary Note 3 and Supplementary Figures 6 and 7 for details). It is worth noting the substantial advancement that this work brings compared to our previous work on nano-electromechanical metasurfaces^27^. The introduction of the asymmetry uniquely enables single mode operation, wide phase tunability without mirror, and wavefront shaping in wavelength scale.

### Fabrication and optical characterization of the active metasurfaces

**Fig. 3:**
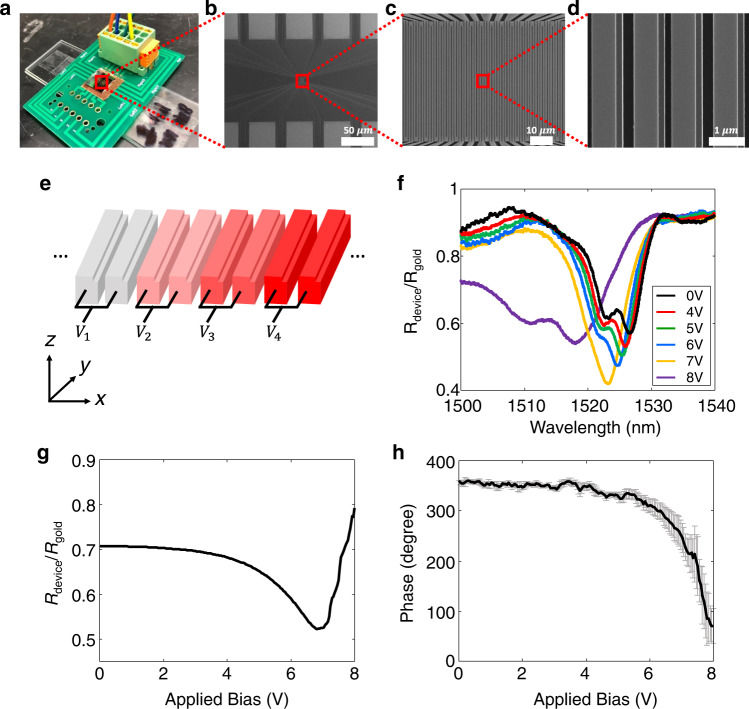
Optical characterization of nano-electromechanical metasurface tuning. **a** Optical image of the fabricated metasurface. Electrodes in the device are wire-bonded to a custom-printed circuit board. **b**–**d** Scanning electron microscopy images of the metasurface. Every pair of nanostructure is connected to the electrodes. Scale bars in **b**–**d** denote 500, 10, and 1 μm, respectively. **e** Schematic of electrical configuration. Four different electrical biases, *V*_1_, *V*_2_, *V*_3_, and *V*_4_, are periodically applied to every four pairs of the nanostructures. **f**–**h** *V*_2_ = *V*_4_ and *V*_1_ = *V*_3_ = 0. **f** Measured reflection spectra for TE-polarized normally incident light. The spectra are measured under six different biases (see legend) and plotted in different colors. **g** Measured intensity modulation under different biases at the wavelength of 1524 nm. The applied bias varies from 0 V to 8 V. **h** Measured phase shift of the metasurface at the wavelength of 1524 nm as a function of the applied biases from 0 V to 8 V. Error bars represent standard deviations of the estimated phase shifts.

We fabricate the active metasurface using a standard silicon-on-insulator wafer and sequential nanofabrication process (see Methods for details). Figure 3a shows a photographic image of the device. In Fig. 3a, the device is wire-bonded to a custom-made printed circuit board for connections to external electrical sources. Scanning electron microscope images of the devices are shown in Fig. 3b–d. In Fig. 3d, the fabricated asymmetric nanostructures show good agreement with the design shown in Fig. 1a. Figure 3e illustrates the electrical configuration of the device, showing that every four pairs of nanostructures are connected to four different electrodes. In Fig. 3e, *V*_1_, *V*_2_, *V*_3_, and *V*_4_ denote the four different applied biases. The voltage differences between the neighboring nanostructures locally determine the gap sizes. The electrical configuration enables the nano-electromechanical modulation with a periodicity of 4. In other words, four pairs of nanostructures are nano-electomechanically modulated in a periodic manner.

We first characterize the tunable optical properties by implementing the scheme shown in Fig. 2a. While *V*_1_ and *V*_3_ are grounded, *V*_2_ and *V*_4_ are connected to the same external biases. When the TE-polarized light is normally incident, the reflection spectra are measured under different external biases and plotted in Fig. 3f (see Methods and Supplementary Figure 8 for details). Without any bias, the resonance dip was observed around 1526 nm in Fig. 3f, in agreement with the simulated resonance dip at 1529 nm shown in Fig. 2b. The small deviation may result from slight errors in fabrication. When the bias changes from 4V to 8V, blue-shift of the resonances and decrease of the minimum reflection are observed in Fig. 3f, showing great agreement with the simulated results in Fig. 2b. As an objective lens in the setup cannot capture the diffraction at ~44^∘^, the decrease of minimum reflection in Fig. 3f can be explained by the increase of the ±1st-order diffractions at ~44^∘^ (see Supplementary Figure 5 for details).

We experimentally investigate electrical modulations of reflection and reflected phase, also implementing the scheme shown in Fig. 2a. To verify the intensity modulation, the reflection is measured at 1524 nm by increasing the applied bias from 0 to 8V, and as shown in Fig. 3g the minimum reflection is higher than 50% (see Supplementary Figure 9 for measured intensity modulations at different wavelengths). In addition, we measure the phase modulation at 1524 nm using a Michelson-type interferometer setup^27^ (see Methods and Supplementary Figure 8 for details). Figure 3h shows measured phase shifts as a function of the applied bias, with over 289.6^∘^ shift for 8V.

### Experimental demonstration of electrically controllable diffraction

**Fig. 4:**
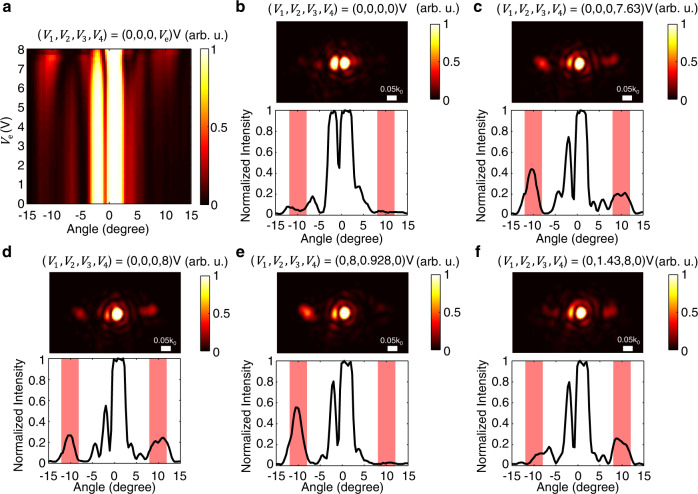
Tunable diffraction with the asymmetric nano-electromechanical metasurface. **a** Measured intensity at the Fourier plane of the metasurface as a function of the applied bias from 0 V to 8 V (*λ* = 1524 nm). At each bias, the intensity is normalized by the peak intensity near 0^∘^. The ±1st order diffractions start to appear ~±10^∘^ when the applied bias is over 6 V. On top of the image, the values of *V*_1_–*V*_4_ are noted and only *V*_4_ is changed. **b**–**f** Measured diffraction patterns for various configurations of the applied biases (*λ* = 1524 nm). (top) Normalized intensity images are measured at the Fourier plane of the metasurface. The values of *V*_1_–*V*_4_ are noted on top of the images. Scale bars are 0.05*k*_0_ where *k*_0_ is a magnitude of wave vector in free-space. (bottom) Measured cross-sectional intensity profiles are plotted as a function of the diffraction angle. The intensities are normalized by the peak intensity at 0^∘^. The diffracted signals ~±10^∘^ are denoted by red shades.

After validating the wide phase tunability and high reflection, we demonstrate electrical control of the diffraction patterns. As shown in Fig. 3e, the device has a fixed periodicity of 4, and it realizes beam deflections into the ±1st orders and beam deflection into the +1st or −1st order with the diffraction angle of ±10^∘^. While *V*_1_ is connected to ground, the values of *V*_2_, *V*_3_, and *V*_4_ are controlled to verify the dynamic diffraction patterns. We image a Fourier plane of the metasurface such that the diffraction patterns are directly measured (see Methods and Supplementary Figure 8 for details). First, we only increase *V*_4_ from 0 to 8V continuously and observe the diffraction intensity changes in the Fourier plane. In Fig. 4a, the diffraction occurs near ±10^∘^ for the large bias over 6V. As shown in Fig. 4a, b, negligible signals are observed at 10^∘^ when no bias is applied. The strongest diffraction intensity is observed when *V*_4_ is at 7.63V (Fig. 4a, c), with the −1st order signal that is stronger than the +1st order. This asymmetric diffraction mainly results from the inherent asymmetry of the structure, showing agreement in the numerical results shown in Supplementary Figures 5–7. Interestingly, when the *V*_4_ further increases up to 8V, the devices achieve comparable ±1st order diffractions in Fig. 4d so nearly symmetric beam deflection into the ±1st order is realized. Next, we control *V*_2_, *V*_3_, and *V*_4_ to demonstrate beam deflection into either the −1st or +1st order diffraction. In Fig. 4e, the device achieves strong −1st order diffraction with normalized intensity reaching 55.7%. Compared to the results shown in 4c, the +1st order diffraction is well suppressed in 4e and its normalized intensity is as small as 3.13%. Likewise, the device can provide strong +1st order diffraction by adjusting the electrical bias as shown in Fig. 4f. In addition to the electrical diffraction control, we should mention that the lobes near 0^∘^ in Fig. 4a–e are split. The degradation may stem from imperfect fabrication and finite size effect. Nonetheless, we can readily move the resonance away from the operating wavelength via electrostatic forces and the artifacts in the large lobe near 0^∘^ vanish (see Supplementary Figure 10 for details). We should also note that such degradation has not been observed in ±1st diffraction order. For most of applications, the quality of the +1st and −1st order diffractions are important.

The strong 0th order signals are observed in Fig. 4a–f, indicating that the diffraction efficiencies are not as efficient as the numerical results shown in Supplementary Figures 6 and 7. We expect that the low experimental efficiency mainly results from imperfect fabrications and the finite size effect. To demonstrate more efficient devices, we fabricate another array of the metasurfaces with changes in the electrical configuration (see Supplementary Figure 11 for details). The best device shows the +1st and −1st order diffractions with normalized intensities 6.05 dB and 3.75 dB larger than the 0th order diffraction, respectively (see Supplementary Figure 11 for details). It experimentally points out that further optimization in the nanofabrication process improves the efficiency of the device.

## Discussion

We utilized the asymmetric dielectric metasurfaces for the realization of tunable phase SLMs, revealing that asymmetric radiation is the key characteristic for designing reflective SLMs without mirrors. The asymmetric metasurfaces not only have shown interesting physical properties such as a strong single-sided phase response, but also have offered practical advantages. For example, it uniquely has allowed for the use of standard silicon-on-insulator wafers in which mirrors are usually not included. Furthermore, the strong phase response can be modulated by not only NEMS, but various active mechanisms for all-solid-state active metasurfaces^34,37–39^. Such all-solid-state active metasurfaces are expected to overcome several limits of mechanical systems such as fragility and limited aperture size.

Here we employed two sequential nanofabrication processes to create asymmetric nanostructures shown in Fig. 3d (see Methods for details). The complexity of the multi-layer nanofabrication may hinder the scalable production of the proposed devices. However, we envision that slanted gratings, which can also achieve asymmetric radiation patterns^35,36^ can replace the proposed structures for scalable production as they can be fabricated with a single lithography step and angled etching techniques.

In summary, we experimentally demonstrated nano-electromechanically tunable phase SLMs enabled by asymmetric metasurfaces. The active metasurfaces numerically and experimentally achieved wide phase tunability, high absolute reflection, and wavelength-scale pixel size. Finally, we demonstrated the nano-electromechanical control of the diffraction patterns. In general, this work experimentally showcases the potential of the asymmetric resonant dielectric metasurfaces for applications in the next-generation SLMs.

## Methods

### Simulation and design

The reflected spectra are calculated using the rigorous coupled wave analysis technique^40^. Assuming infinite lengths for the silicon nanostructures, 2D simulations were performed. While we assume that the silicon structures are surrounded by air in Fig. 1a, a 700-nm air gap, a 2300-nm thick silicon oxide layer, and a silicon substrate are added underneath the silicon structure in Fig. 2 to simulate the fabricated devices. The eigenmode analysis shown in Figs. 1d, S1, and S3 are performed using commercial software based on the finite elements method, COMSOL®. Refractive indices of Si and SiO_2_ for the telecom wavelength in the simulation are 3.4 and 1.45, respectively.

### Device fabrication

We use a silicon-on-insulator wafer with a device layer of 1500 nm and a buffered oxide layer of 3 μm on a 1 mm thick silicon substrate. First, the device layer is thinned down to the target thickness of ~838 nm using reactive-ion-etching with a gas mixture of SF_6_ and C_4_F_8_. The nanofabrication includes three sequential electron beam lithography steps, the first one for the grating structures, the second one for the notches, and the last one for the electrodes. For all electron beam lithography steps, a ~300-nm-thick positive electron resist (ZEP-520A, Zeon) is spin-coated on the device. The patterns are generated by 100 kV electron beam exposure (EBPG5200, Raith GmbH), and the resist is developed in a developer solution (ZED-N50, Zeon). To pattern the gratings and notches, the ZEP resist is utilized as a soft mask in the reactive-ion etching steps and then removed by remover PG (Microchem). After the fabrication of the aysmmetric silicon nanostructures, the electrodes were patterned by electron beam lithography, the deposition of chrome and gold (5 nm and 65 nm) layers, and liftoff. To etch the buffered oxide layer under the gratings, we exploit buffered hydrofluoric acid. Like the under-cut process in^27^, the time of the under-cut process is adjusted carefully such that the anchors are supported by the SiO_2_ while the nanostructures are fully suspended. After the under-cut, the device is dried by a critical point dryer. After the drying process, severe out-of-plane or in-plane deformation of the nanostructures has not been observed under the SEM images. Finally, the device is connected to a custom-printed circuit board using a wire bonder (WestBond 7476D). The fabricated metasurface shown in Fig. 3b–d consists of 36 pairs of asymmetric suspended nanostructures. In this paper, all of the nanostructures are 50 μm long in *y* axis and both ends of the nanostructures are connected to either anchors or large silicon layers that are supported by the buffered oxide layers^27^. Furthermore, *g*_1_ and *g*_2_ are adjusted in the fabrication process to make *g*_1_ 120 nm smaller than *g*_2_ such that the nano-electromechanical tuning leads to efficient tuning of the resonance. As shown in Fig. 3e, the device has four different sets of electrodes. The customized PCB is capable of providing four independent voltages to the sets of electrodes in the device. The independent biases are produced by Arduino (Arduino Uno R3). Specifically, four different pulse width modulation (PWM) channels in Arduino are connected to four PWM to DC converter modules (LC-LM358-PWM2V) and a custom external circuit. As a result, the applied biases are individually controllable by updating the four PWM channels, which are programmed via a common laptop by using the Arduino software (IDE).

### Measurement procedure

All of the measurements presented in this paper are characterized using the set-ups shown schematically in Supplementary Figure 8^27,34^. We use a tunable laser (Photonetics, TUNICS-Plus) as the light source. A beam splitter is placed in front of the fiber collimator (Thorlabs, F260FC-1550) to capture the power from the source and send the light to the sample. For reference, the power from the source is measured by an InGaAs detector (Thorlabs, PDA10CS). A polarized beam splitter (PBS), a half waveplate, and a polarizer are inserted to set the polarized state of the incident light to TE polarization. The sample at the object plane is imaged by a ×20 infinity-corrected objective lens (Mitutoyo, M Plan Apo NIR) and a tube lens with a focal length of 200 mm. The position of the tube lens and the mounting stage of the sample is adjusted to ensure normal incidence. At the image plane, an iris (Thorlabs, ID25) is inserted to select a region of interest with a diameter of 45 μm in the object plane. The spatially filtered light was either focused onto another InGaAs detector for the measurement of the spectra, or imaged on an InGaAs SWIR camera (Goodrich, SU320HX-1.7RT) using relay optics. All reflection signals were obtained by dividing the signal from the sample by the signal from the sources. Due to different input polarization states, the incident power onto the sample varies at different wavelengths. Thus, the signals are further normalized by the signals from the gold. To measure the phase response shown in Fig. 3h, we use a Michelson-type interferometer setup^27^. A part of the setup marked by a black dashed box in Supplementary Fig. 8 is only utilized for the phase measurement. As the field of view of the objective lens is larger than the device size, the input light illuminates the metasurface and unpatterned regions at the same time and forms fringes with a reference beam at the image plane. The phase shift is mainly evaluated by the shift of the fringes on the metasurface, while we ensure that the fringes on the unpatterned regions are unchanged^27^. Also, the estimated phases shown in Fig. 3h are averaged over 48 measurements using the same device, and error bars represent standard deviations of the estimated values.

To measure the diffracted signal shown in Fig. 4 and Supplementary Figure 10, we image a Fourier plane of the metasurface^27^. Parts of the setup marked by a green solid box in Supplementary Figure 8 are only utilized for the diffraction pattern measurement. Specifically, relay lenses, L6 and L7, and a flip mirror are used to image the Fourier plane with the InGaAs SWIR camera. Also, we built a custom microscope setup at the wavelength of 850 nm to align the sample when we image the Fourier focal plane.

## Supplementary information

Supplementary information

## Data Availability

The main dataset generated in this study has been deposited in CaltechData under accession code: 10.22002/D1.20291.

## Code availability

The main codes generated in this study have been deposited in CaltechData under accession code: 10.22002/D1.20291.
