# Supplementary material for: Nano-electromechanical spatial light modulator enabled by asymmetric resonant dielectric metasurfaces
Source: Nat Commun. 2022 Oct 3;13:5811. doi: 10.1038/s41467-022-33449-9 (PMC9530114; doi:10.1038/s41467-022-33449-9)
Supplement: Supplementary file 1 — Supplementary information [file 41467_2022_33449_MOESM1_ESM.pdf]

Supporting information: Nano-electromechanical spatial light modulator enabled by asymmetric resonant dielectric metasurfaces

Hyounghan Kwon,[1,2] Tianzhe Zheng,[1] and Andrei Faraon[1,2,*]

[1]*T. J. Watson Laboratory of Applied Physics and Kavli Nanoscience Institute, California Institute of Technology, 1200 E. California Blvd., Pasadena, CA 91125, USA*

[2]*Department of Electrical Engineering, California Institute of Technology, 1200 E. California Blvd., Pasadena, CA 91125, USA*

[*] Corresponding author: A.F.: faraon@caltech.edu

SUPPLEMENTARY NOTE1: TEMPORAL COUPLED-MODE THEORY FOR ASYMMETRIC RESONANT DIELECTRIC METASURFACES

For resonant metasurfaces or photonic crystals, it is known that the temporal response of the resonator can be described by temporal coupled mode theory (TCMT) [1, 2]. As seen in Fig. **1b** in the main text, with normally incident light, the resonant metasurface can be modeled by a single-mode resonator that is coupled to two ports. The dynamics of the optical resonance can be generally formulated by:

$$\frac{du}{dt} = (iw_0 - \frac{1}{\tau_1} - \frac{1}{\tau_2} - \frac{1}{\tau_{nr}})u + \begin{pmatrix} d_1 & d_2 \end{pmatrix} \begin{pmatrix} s_1^+ \\ s_2^+ \end{pmatrix}, \quad \textbf{(S1)}$$

$$\begin{pmatrix} s_1^- \\ s_2^- \end{pmatrix} = C \begin{pmatrix} s_1^+ \\ s_2^+ \end{pmatrix} + \begin{pmatrix} d_1 \\ d_2 \end{pmatrix} u, \quad \textbf{(S2)}$$

where u and w_0 correspond to complex amplitude of the resonance and the central resonance frequency, respectively; d_1 and d_2 are the coupling coefficients between the two ports and the resonances; the resonance radiatively decays into port 1 and 2 with decay rates of $\frac{1}{\tau_1}$ and $\frac{1}{\tau_2}$, respectively; $\frac{1}{\tau_{nr}}$ is the nonradiative decay rate; s_1^+ (s_1^-) and s_2^+ (s_2^-) are amplitudes of the incoming (outgoing) waves from the ports; C is a direct-transport scattering matrix, written by $C = e^{i\phi_d} \begin{pmatrix} r & it \\ it & r \end{pmatrix}$, where r, t, and ϕ_d are the real reflection coefficient, the real transmission coefficient, and the phase factor, respectively. C generally describes the direct coupling between the incoming and outgoing waves. In addition, as ϕ_d depends on the selection of reference planes in the model, ϕ_d can be set to 0 for simplicity. In addition, we here assume that $\frac{1}{\tau_{nr}}$ is negligible because silicon is almost lossless in the telecom wavelength range. According to the time-reversal symmetry and the energy conservation, the coupling coefficients satisfy

$$d_1^* d_1 = \frac{2}{\tau_1}, \qquad d_2^* d_2 = \frac{2}{\tau_2}, \quad \textbf{(S3)}$$

$$C \begin{pmatrix} d_1^* \\ d_2^* \end{pmatrix} = - \begin{pmatrix} d_1 \\ d_2 \end{pmatrix}, \quad \textbf{(S4)}$$

revealing that the coupling conditions are fundamentally related to the decay rates of the resonances as well as the direct-transport scattering [2, 3].

According to Refs. [3, 4], we could analytically solve Eqs. S1-S4 to obtain the Eqs. **1** and **2** in the main text. In detail, when the system is driven by a continuous laser, whose frequency is w, we can derive u as a function of w from Eq. S1

$$u = \frac{\begin{pmatrix} d_1 & d_2 \end{pmatrix}\begin{pmatrix} s_1^+ \\ s_2^+ \end{pmatrix}}{i(w - w_0) + \frac{1}{\tau_{tot}}}, \tag{S5}$$

where $\frac{1}{\tau_{tot}} = \frac{1}{\tau_1} + \frac{1}{\tau_2}$. By inserting Eq. S5 into Eq. S2, the outgoing waves can be described by,

$$\begin{pmatrix} s_1^- \\ s_2^- \end{pmatrix} = C\begin{pmatrix} s_1^+ \\ s_2^+ \end{pmatrix} + \frac{\begin{pmatrix} d_1 \\ d_2 \end{pmatrix}\begin{pmatrix} d_1 & d_2 \end{pmatrix}}{i(w - w_0) + \frac{1}{\tau_{tot}}}\begin{pmatrix} s_1^+ \\ s_2^+ \end{pmatrix}. \tag{S6}$$

From Eq. S6, we can derive the reflection spectra of port 1 and 2, r_1 and r_2:

$$r_1 = \left.\frac{s_1^-}{s_1^+}\right|_{s_2^+=0} = r + \frac{{d_1}^2}{i(w - w_0) + \frac{1}{\tau_{tot}}}, \tag{S7}$$

$$r_2 = \left.\frac{s_2^-}{s_2^+}\right|_{s_1^+=0} = r + \frac{{d_2}^2}{i(w - w_0) + \frac{1}{\tau_{tot}}}. \tag{S8}$$

Next, Eqs. S3 and S4 are employed to eliminate phase ambiguity of d_1 and d_2 in Eqs. S7 and S8. Specifically, from Eq. S3, d_1 and d_2 can be described by

$$d_1 = \sqrt{\frac{2}{\tau_1}}e^{i\theta_1}, \quad d_2 = \sqrt{\frac{2}{\tau_2}}e^{i\theta_2}, \tag{S9}$$

where θ_1 and θ_2 are phases of the coupling coefficients of d_1 and d_2, respectively. By inserting Eq. S9 into Eq. S4, $cos(2\theta_1)$ and $cos(2\theta_2)$ can be derived by

$$\cos(2\theta_1) = \frac{\tau_1}{2r}\left(- \frac{r^2}{\tau_{tot}} - \frac{1}{\sigma}\right), \tag{S10}$$

$$\cos(2\theta_2) = \frac{\tau_2}{2r}\left(- \frac{r^2}{\tau_{tot}} + \frac{1}{\sigma}\right), \tag{S11}$$

where $\frac{1}{\sigma} = \frac{1}{\tau_1} - \frac{1}{\tau_2}$. Then, $sin(2\theta_1)$ and $sin(2\theta_2)$ are expressed as:

$$\sin(2\theta_1) = \pm\frac{\tau_1}{2r}\sqrt{\frac{4r^2}{{\tau_1}^2} - \frac{r^4}{{\tau_{tot}}^2} - \frac{1}{\sigma^2} - \frac{2r^2}{\tau_{tot}\sigma}}, \quad \textbf{(S12)}$$

$$\sin(2\theta_2) = \pm\frac{\tau_2}{2r}\sqrt{\frac{4r^2}{{\tau_2}^2} - \frac{r^4}{{\tau_{tot}}^2} - \frac{1}{\sigma^2} + \frac{2r^2}{\tau_{tot}\sigma}}. \quad \textbf{(S13)}$$

As r_1 and r_2 in Eqs. S7 and S8 can be described by $\cos(2\theta_1)$, $\cos(2\theta_2)$, $\sin(2\theta_1)$, and $\sin(2\theta_2)$, we can derive the Eqs. **1** and **2** in the main text:

$$r_1 = r + \frac{\frac{2}{\tau_1}\Big(\cos(2\theta_1) + i\sin(2\theta_1)\Big)}{i(w-w_0) + \frac{1}{\tau_{tot}}} = \frac{i\left[r(w-w_0)\pm\sqrt{\frac{2}{\tau_1^2} + \frac{2}{\tau_2^2} - \frac{r^2}{\tau_{tot}^2} - \frac{1}{r^2\sigma^2}}\right] - \frac{1}{r\sigma}}{i(w-w_0) + \frac{1}{\tau_{tot}}}, \quad \textbf{(S14)}$$

$$r_2 = r + \frac{\frac{2}{\tau_2}\Big(\cos(2\theta_2) + i\sin(2\theta_2)\Big)}{i(w-w_0) + \frac{1}{\tau_{tot}}} = \frac{i\left[r(w-w_0)\pm\sqrt{\frac{2}{\tau_1^2} + \frac{2}{\tau_2^2} - \frac{r^2}{\tau_{tot}^2} - \frac{1}{r^2\sigma^2}}\right] + \frac{1}{r\sigma}}{i(w-w_0) + \frac{1}{\tau_{tot}}}. \quad \textbf{(S15)}$$

The two-port resonator model shown in Fig. **1b** in the main text generally describes any single-mode resonant metasurfaces under normal incidence. When the light is obliquely incident and the metasurface is in sub-wavelength regime (i.e. there is no diffraction), the metasurface can be modeled by a four-port resonator. The general description of the four-port resonator model can be found in Ref. [4]. Here, we only deal with a fully symmetric case where the resonance equally decays into the four ports with the decay rate of $\frac{1}{\tau_0}$. The reflection spectrum of the symmetric resonator, r_s, can be expressed as [4]:

$$r_s = \frac{i\left[r(w-w_0)\pm\frac{2}{\tau_0}\sqrt{1-r^2}\right]}{i(w-w_0) + \frac{2}{\tau_0}}. \quad \textbf{(S16)}$$

We should note that Eq. S16 becomes identical to Eq. S14 or S15 when $\frac{1}{\tau_1} = \frac{1}{\tau_2} = \frac{1}{\tau_0}$. In other words, if the structures are symmetric with respect to all available ports and $\frac{1}{\tau_0} \neq 0$, the single-mode resonance is always critically coupled to the excitation.

SUPPLEMENTARY NOTE2: NUMERICAL INVESTIGATIONS ON SYMMETRIC RESONANT DIELECTRIC METASURFACES

To physically implement a symmetrical case of the theoretical model in Supplementary Note 1, we simulate the metasurfaces possessing the mirror symmetry in the z-direction. In Fig. S4**a**, the metasurface grating is composed of Si nanobars and surrounded by air. Specifically, the 841 nm wide and 838nm thick 2D nanostructures are periodically arranged with the lattice constant of 1093 nm. Figure S4**b** shows an electrical field profile of the TE-polarized eigenmode at Γ point. The mode in Fig. S4**b** originates from the Mie mode hosted by individual Si nanostructures (see Fig. S1 for details). Reflection and reflected phase spectra are calculated under 0° and 5° tilted incident lights and plotted in Figs. S4**c** and S4**d**. The resonance is not coupled to the normally incident light in Figs. S4**c** and S4**d**. That is due to the symmetry mismatch between the excitation and the eigenmode. In detail, the plane-wave excitation and the eigenmode in Fig. S4**b** are odd and even under C_2 rotation, respectively. In contrast, the 5° tilted light excites the resonant mode by breaking the odd symmetry of the incident light. However, the critical coupling between the excitation and the resonant mode occurs, causing negligible reflection at the resonance in Fig. S4**c** and limited phase shifts smaller than 180° in Fig. S4**d**. Finally, the results shown in Figs. S4**c** and S4**d** agree with the previous discussion of the symmetric resonator in Supplementary Note 1.

SUPPLEMENTARY NOTE3: NUMERICAL INVESTIGATIONS ON BEAM STEERING OF THE ASYMMETRIC METASURFACES

Here, we numerically verify the metasurfaces' capability of beam steering, using a pair of the nanostructure as a building block of the proposed active metasurfaces. Specifically, the gaps of the pairs of nanostructure are adjusted by the applied biases such that the metasurface manipulates the wavefronts of the reflected light. When assuming that the phase is locally determined by the gap of the two nanostructures, we can exploit the relationship between ϕ_{0th} and $\frac{g_1-g_2}{2}$ plotted in Fig. **2d** in the main text as a lookup table to design the metasurfaces. In other words, once the desired phase distribution is determined, the gaps of nanostructures can be inversely obtained from Fig. **2d** in the main text. It is noteworthy to mention that this lookup table approach is widely used in passive and active metasurfaces. First, we investigate a blazed diffraction grating of which the linear phase gradient is negative. As shown in Fig. S6**a**, the period of the grating, p_g, is determined by periodicity and 2Λ, where the periodicity represents the number of the pairs in one period of the blazed grating. As the blazed grating is designed to have the negative phase gradient of $-\frac{2\pi}{p_g}$, the metasurfaces expect to cause the dominant -1st order diffraction at the angle of $-\theta_g = -\sin^{-1}(\frac{\lambda}{p_g})$. We simulate negative phase gradient gratings having periodicity of 4 and 6. The spectra of reflected power coefficients for the 0th and $\pm$1st and order diffractions are plotted in Figs. S6**b** and S6**c**. At 1529 nm, the reflected power coefficients of the -1st order diffraction are 16.7 and 27.0% in Figs. S6**b** and S6**c**, respectively. In contrast, the calculated reflected power coefficients of the $+1$st (0th) order diffraction are 2.53% (5.69%) and 4.66%(7.47%) in Figs. S6**b** and S6**c**, respectively. Similarly, the blazed gratings with positive phase gradients are also investigated. In Fig. S6**d**, the arrangement of the gap sizes are simply reversed compared to the arrangement of the negative phase gradient blazed gratings shown in Fig S6**a**. Then, the reversed nanomechanical displacements realize the positive phase gradient of $\frac{2\pi}{p_g}$, expecting to result in the dominant $+1$st order diffraction at the angle of θ_g. The reflected power coefficient spectra of the positive phase gradient blazed gratings are plotted in Figs. S6**e** and S6**f**. The dominant $+1$st order diffraction and the suppressed 0th and -1st order diffractions are observed in Figs S6**e** and S6**f**. At the design wavelength of 1529 nm, the reflected power coefficients of the $+1$st order diffraction are 10.1% and 18.0% in Figs. S6**e** and S6**f**, respectively. At the same wavelength, the calculated reflected power coefficients of the -1st (0th) diffraction order are 4.26% (7.46%) and 7.11%(8.49%) in Figs. S6**e** and S6**f**, respectively. For both periodicities of 4 and 6, the negative phase-gradient gratings used in Figs. S6**b** and S6**c** perform

more efficiently than the positive phase-gradient gratings used in Figs. **S6e** and **S6f**. The same trend can be also found in the case of periodicity of 1 (see Supplementary Figure **5** for details). We expect that these differences inherently result from the asymmetry of the structure with respect to x-axis. Besides, at the design wavelength of 1529 nm, the reflected power coefficients of all available diffraction orders are plotted in Fig. **S7**, showing that all high-order diffraction components are suppressed compared to the desired diffraction order. In particular, it is worth noting that the highest-order diffractions at the angle $\pm 44°$ are well suppressed when the periodicity is extended over 1.

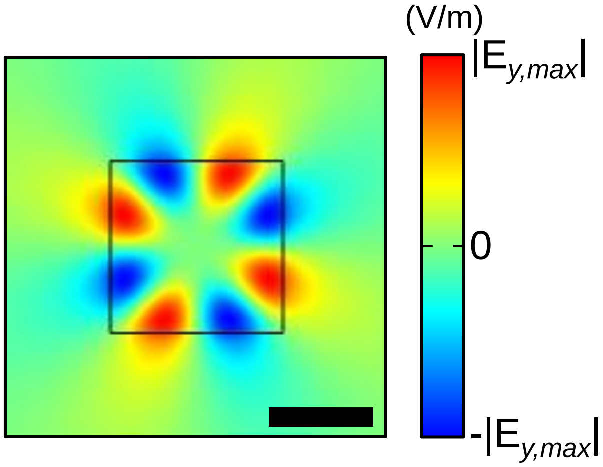

Figure S1 Electric field profile of high-order Mie mode resonance in a symmetric Si nanobar. *y*-components of electric fields are plotted. The eigenmode is found without the periodic boundary condition. The width and thickness are 841 and 838 nm, respectively. The calculated complex eigenfrequency is (191 + i0.291) THz, corresponding to the resonant wavelength of 1569 nm and Q-factor of 328. Scale bar denotes 500 nm.

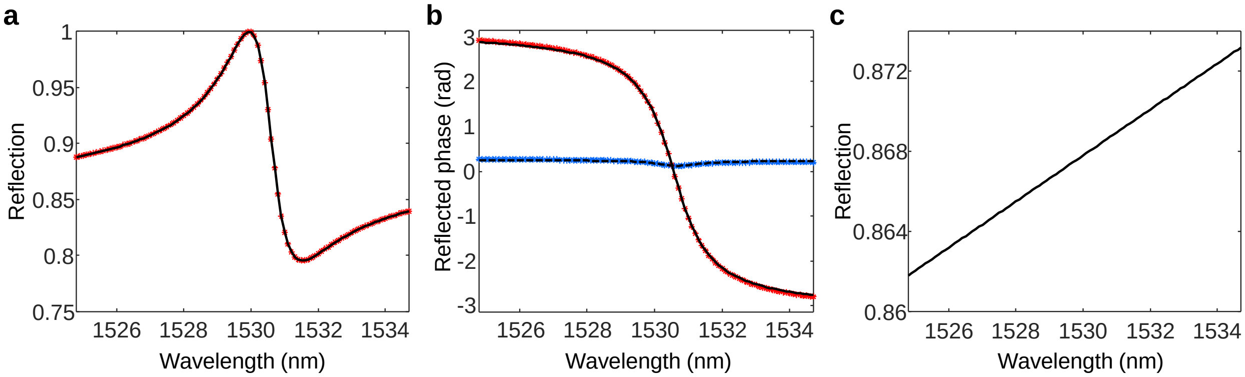

Figure S2 Analytical fitting of calculated reflection and reflected phase spectra of the asymmetric metasurface. **a** and **b** Numerical data shown in Figs. **1e** and **1f** in the main text is fitted by using Eqs. **1** and **2** in the main text. As shown in Fig. **1c** in the main text, $|r_{TE1}|^2$ ($|r_{TEw}|^2$) and ϕ_{TE1} (ϕ_{TE2}) represent reflection and reflected phase for top (bottom) illumination, respectively. From the fitting, we find the Q-factor and $\frac{\tau_2}{\tau_1}$ of 1004 and 17.52, respectively. **a**: Calculated and fitted spectra of $|r_{TE1}|^2$ and $|r_{TE2}|^2$. Red asterisks show the calculated spectra of $|r_{TE1}|^2$ or $|r_{TE2}|^2$. The fitted spectrum is plotted by a black solid line. **b**: Calculated and fitted spectra of ϕ_{TE1} and ϕ_{TE2}. Red and blue asterisks represent the calculated spectra of ϕ_{TE1} and ϕ_{TE2}, respectively. The fitted spectra of ϕ_{TE1} and ϕ_{TE2} are plotted by solid and dashed black lines, respectively. **c** Fitted reflection spectra of the direct-transport scattering process.

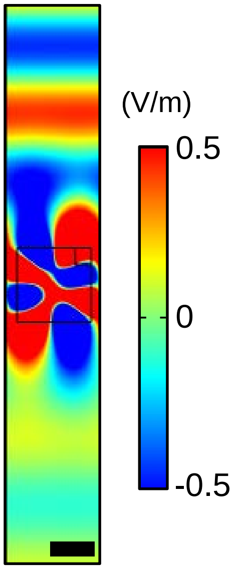

Figure S3 Numerical investigation of asymmetric radiation of the proposed metasurface. Calculated electric field profiles of the eigenmode at Γ point. The *y*-components of the electric field profiles of the eigenmode are plotted. Strong radiation toward top direction is observed. In simulation, the power ratio between the top and bottom radiations and Q-factor of the eigenmode are 17.54 and 1021, respectively.

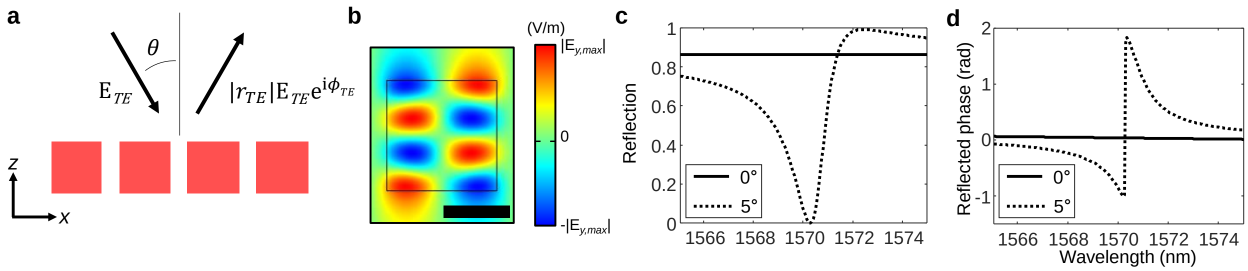

Figure S4 Numerical investigations on resonant reflection behaviors of symmetric metasurfaces. a Schematic illustration of the suspended symmetric metasurfaces. The metasurface possesses mirror symmetry in the z-direction with respect to the middle of the nanostructure. The TE polarized light is incident on the metasurface. **b** Simulated electrical field profile of the eigenmode at Γ point. At the wavelength of 1568 nm, the y-components of the electrical fields are plotted. Scale bar denotes 500 nm. **c** and **d** Calculated reflection and reflected phase spectra of the symmetric metasurfaces. Solid and dashed curves show the spectra for 0° and 5° tilted TE polarized incident light, respectively.

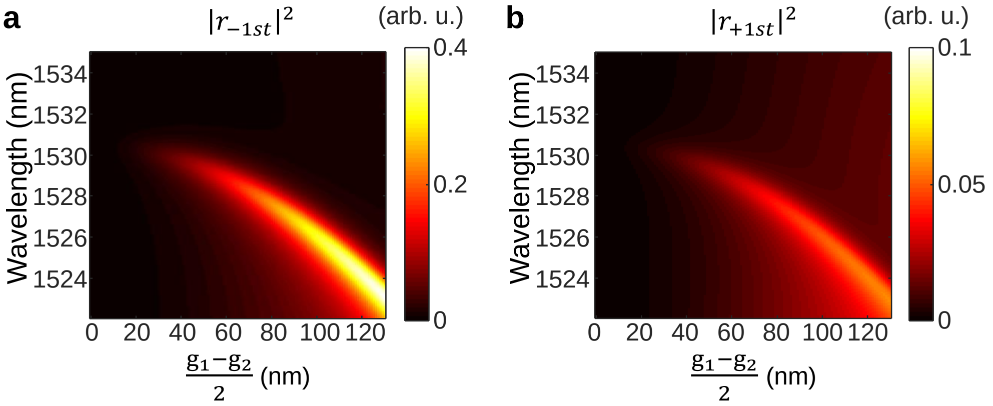

Figure S5 Numerical investigations on high diffraction orders. **a** and **b** Simulated reflected power spectra of the ±1st order diffractions. The reflected power coefficient spectra of the −1st and +1st order diffractions, $|r_{1st}|^2$ and $|r_{+1st}|^2$, are calculated as a function of the nanomechanical tuning, $\frac{g_2-g_1}{2}$, and plotted in **a** and **b**, respectively. It should be noted that the color bars are different in **a** and **b**. The calculated reflected power coefficient spectra of the 0th order diffraction is shown in Fig. **2b** in the main text.

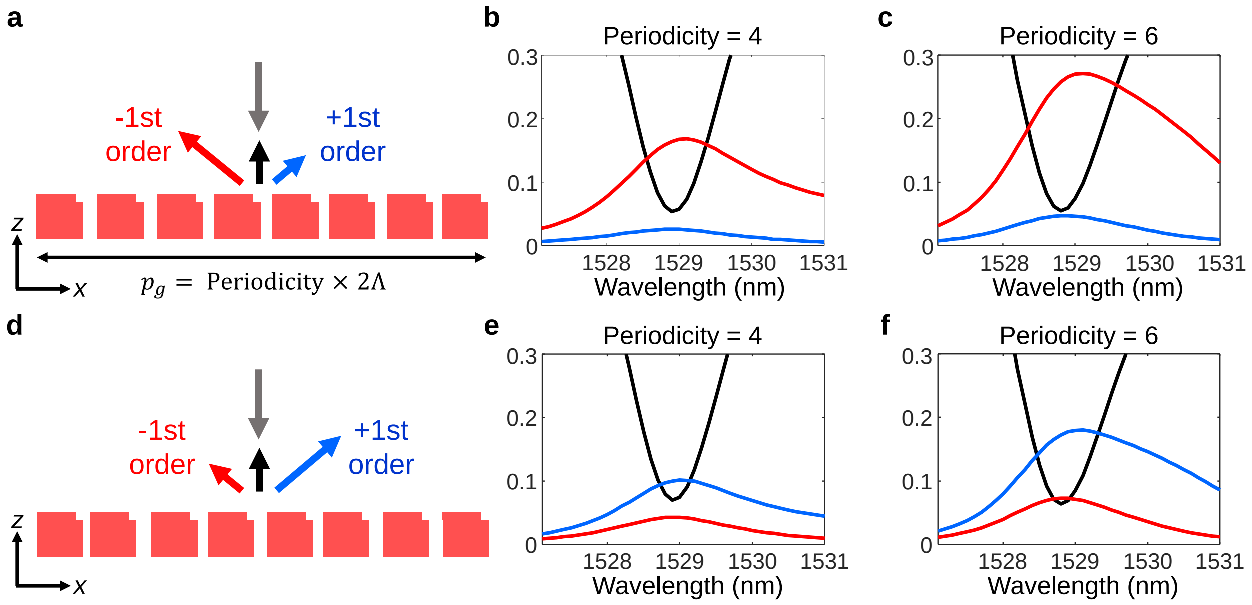

Figure S6 Simulations on nano-electromechanical beam steering. **a** (**d**) Conceptual illustration of nanomechanical beam steering with negative (positive) phase gradients. In reflection, the −1st (1st) order diffraction is dominant over the 0th and +1st (−1st) order diffractions. **b** and **c** (**e** and **f**) Calculated reflected power coefficient spectra of the 0th and ±1st order diffractions where the phase-gradient is negative (postive). The spectra of the 0th, −1st and +1st order diffractions are plotted by black, red, and blue curves, respectively. The periodicities of the metasurface are 4 and 6 pairs in **b** (**e**) and **c** (**f**), respectively.

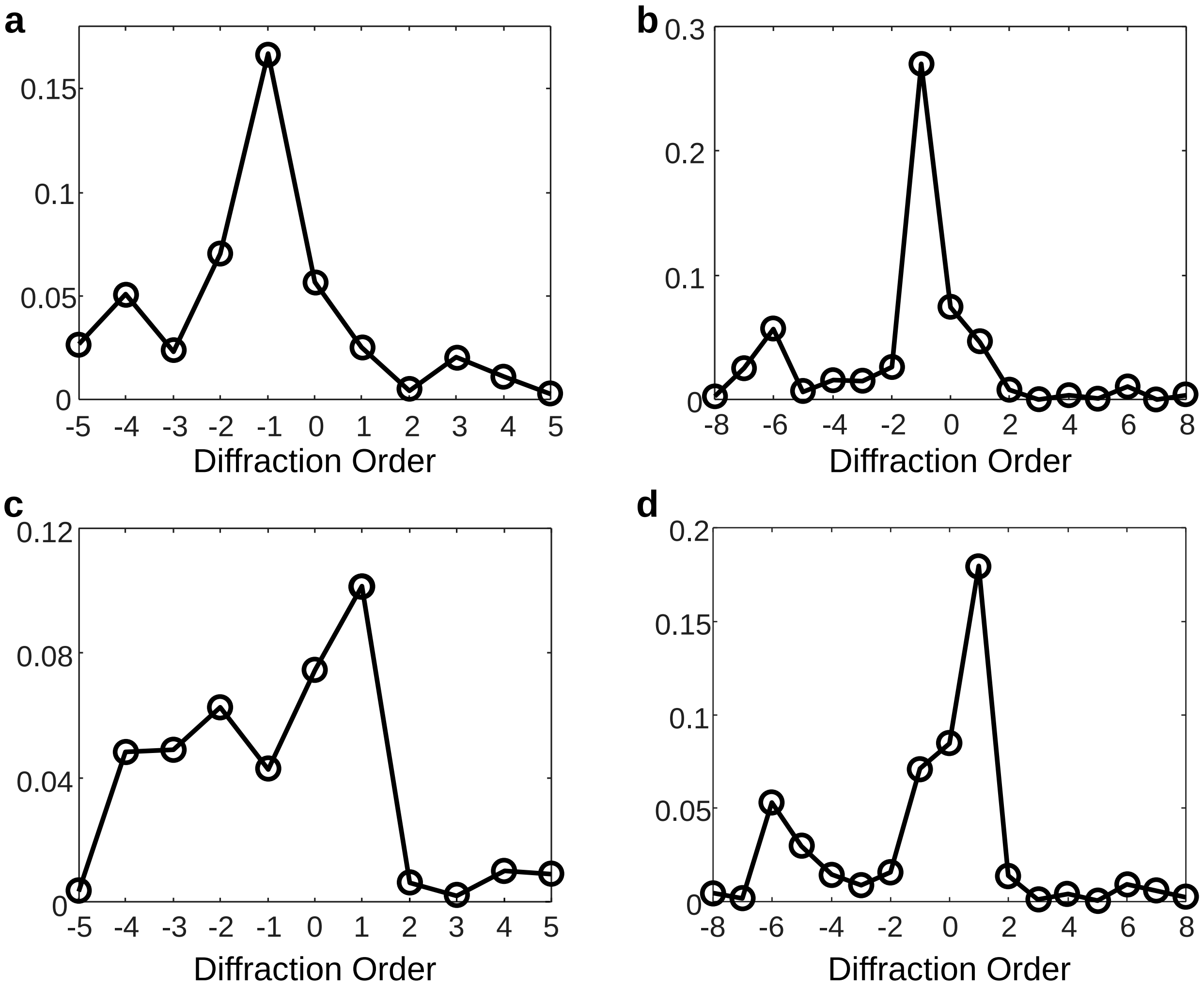

Figure S7 Numerical investigation on reflected power coefficients of the asymmetric metasurface. a-d Calculated reflected power coefficients of the metasurface grating at the design wavelength of 1529 nm. The simulated reflected power coefficients are plotted for all available diffraction orders. The designs used in Figs. S6**b**, S6**c**, S6**e** and S6**f** are employed for **a**, **b**, **c**, and **d**, respectively

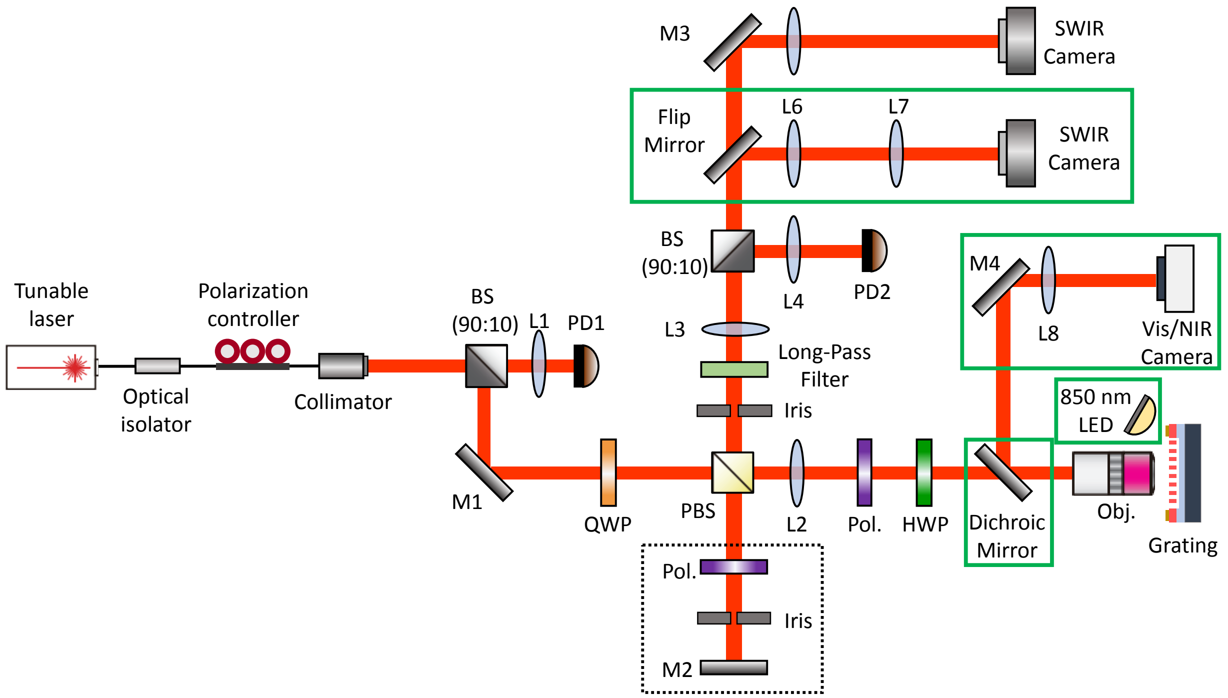

Figure S8 Schematic illustration of the experimental setup. Red lines represent the paths of the light. To achieve the reflection spectra of the TE-polarized input light, Pol. and HWP in front of the objective lens are aligned to 45 and 67.5 degree, respectively. The black dashed box represents optical elements exploited to generate the reference beam for the phase measurement shown in Fig. **3h**. Also, we use optical components in green boxes only for the measurements of the diffraction pattern shown in Figs. **4** and S11. Pol.: linear polarizer. BS: beamsplitter. PBS: polarizing beamsplitter. L: lens. PD: photodetector. M: mirror. QWP: quarter waveplate. HWP: half waveplate. Obj.: microscope objective lens. SWIR camera: short-wave infrared camera.

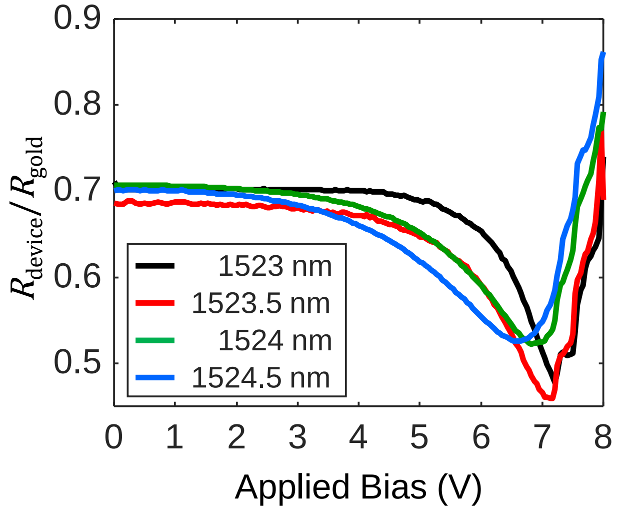

Figure S9 Measured intensity modulations near the resonance wavelength. Intensity modulation is measured at four different wavelengths and plotted in different colors as a function of the applied biases. The applied biases vary from 0V to 8V. The measured wavelength for each color is shown in legend.

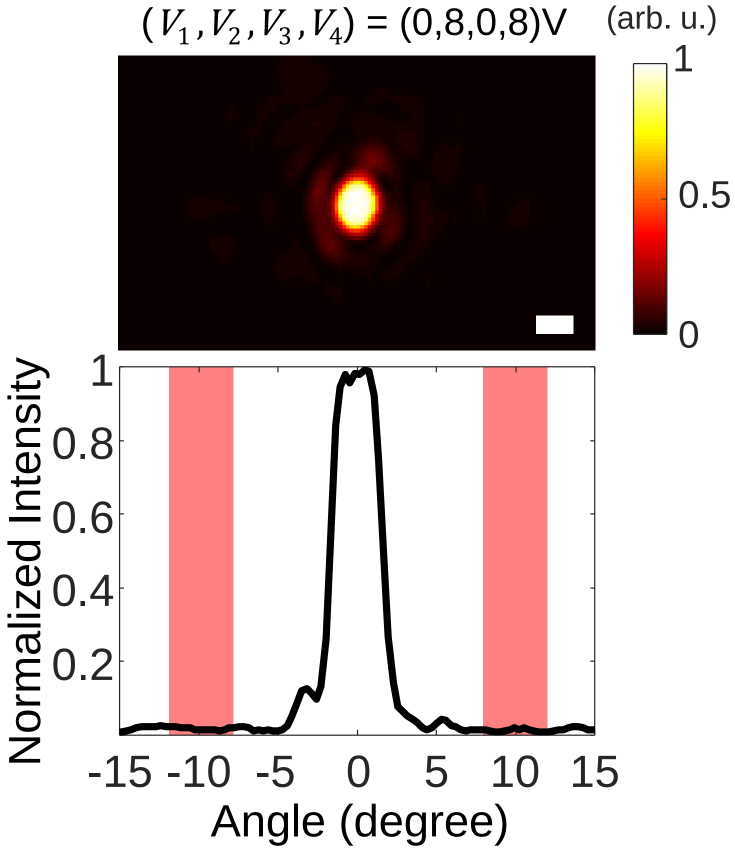

Figure S10 Measured back focal plane image with large resonance shift. Top: The normalized intensity image is measured at the Fourier plane of the metasurface. The values of V_1-V_4 are noted on top of the images. V_2 and V_4 increase up to 8 V to move the resonance below the measured wavelength of 1524 nm. Scale bars are $0.05k_0$ where k_0 is a magnitude of wave vector in free-space. Bottom: The measured cross-sectional intensity profile is plotted as a function of the diffraction angle. The intensities are normalized by the peak intensity at 0°. The diffracted signals near ±10° are denoted by red shades.

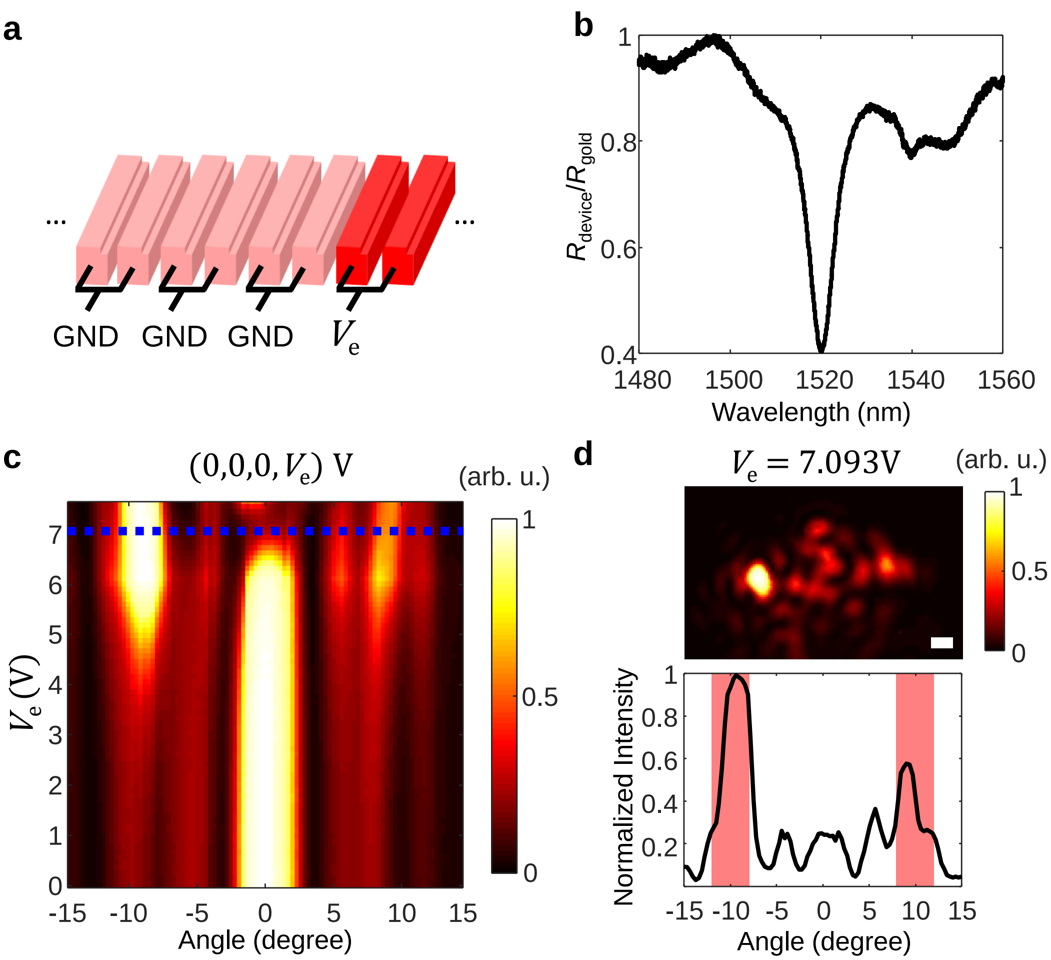

Figure S11 Experimental demonstration of the efficient active metasurface. a Schematic of electrical configuration. With three pairs of the asymmetric nanostructures grounded, only one pair is connected to external bias, V_e, for every four pairs of nanostructures. As two electrodes are required for this electrical bias, a large number of devices can be fabricated and modulated simultaneously [5]. **b** Measured reflection spectrum for TE-polarized normally incident light. The spectrum is measured without any bias and normalized by the reflection from a gold electrode. **c** Measured intensity at the Fourier plane of the metasurface. The intensity is measured at 1519 nm as a function of the applied bias. V_e changes from 0V to 7.6V with a step size of 0.101 V. At each bias, the intensity is normalized by the maximum intensity. **d** Measured diffraction patterns at 1519 nm with V_e of 7.093V. The corresponding data is noted by a blue dashed line in **c**. Top: The normalized intensity image is measured at the Fourier plane of the metasurface. The value of V_e is noted on top of the image. Scale bar denotes $0.05k_0$ where k_0 is a magnitude of wave vector in free-space. Bottom: Measured cross-sectional intensity profile is plotted as a function of the diffraction angle. The intensities are normalized by the peak intensity around $-10°$. The diffracted signals near $\pm10°$ are denoted by red shades. Quantitatively, the -1st and $+1$st order signals are 6.05 dB and 3.75 dB larger than the 0th order signal, respectively

I. REFERENCES

[1] Haus, H. A. *Waves and fields in optoelectronics* (Prentice-Hall, 1984).

[2] Fan, S., Suh, W. & Joannopoulos, J. D. Temporal coupled-mode theory for the fano resonance in optical resonators. *JOSA A* **20**, 569–572 (2003).

[3] Wang, K. X., Yu, Z., Sandhu, S. & Fan, S. Fundamental bounds on decay rates in asymmetric single-mode optical resonators. *Optics letters* **38**, 100–102 (2013).

[4] Zhou, H. et al. Perfect single-sided radiation and absorption without mirrors. *Optica* **3**, 1079–1086 (2016).

[5] Kwon, H., Zheng, T. & Faraon, A. Nano-electromechanical tuning of dual-mode resonant dielectric metasurfaces for dynamic amplitude and phase modulation. *Nano letters* **21**, 2817–2823 (2021).
